# A-to-I RNA Editing in Cancer: From Evaluating the Editing Level to Exploring the Editing Effects

**DOI:** 10.3389/fonc.2020.632187

**Published:** 2021-02-11

**Authors:** Heming Wang, Sinuo Chen, Jiayi Wei, Guangqi Song, Yicheng Zhao

**Affiliations:** ^1^ Clinical Medical College, Changchun University of Chinese Medicine, Changchun, China; ^2^ Department of Gastroenterology and Hepatology, Zhongshan Hospital of Fudan University, Shanghai, China; ^3^ Shanghai Institute of Liver Diseases, Shanghai, China

**Keywords:** ADAR, RNA editing, cancer, non-coding RNA, circular RNAs

## Abstract

As an important regulatory mechanism at the posttranscriptional level in metazoans, adenosine deaminase acting on RNA (ADAR)-induced A-to-I RNA editing modification of double-stranded RNA has been widely detected and reported. Editing may lead to non-synonymous amino acid mutations, RNA secondary structure alterations, pre-mRNA processing changes, and microRNA-mRNA redirection, thereby affecting multiple cellular processes and functions. In recent years, researchers have successfully developed several bioinformatics software tools and pipelines to identify RNA editing sites. However, there are still no widely accepted editing site standards due to the variety of parallel optimization and RNA high-seq protocols and programs. It is also challenging to identify RNA editing by normal protocols in tumor samples due to the high DNA mutation rate. Numerous RNA editing sites have been reported to be located in non-coding regions and can affect the biosynthesis of ncRNAs, including miRNAs and circular RNAs. Predicting the function of RNA editing sites located in non-coding regions and ncRNAs is significantly difficult. In this review, we aim to provide a better understanding of bioinformatics strategies for human cancer A-to-I RNA editing identification and briefly discuss recent advances in related areas, such as the oncogenic and tumor suppressive effects of RNA editing.

## Introduction

**Figure 1:**
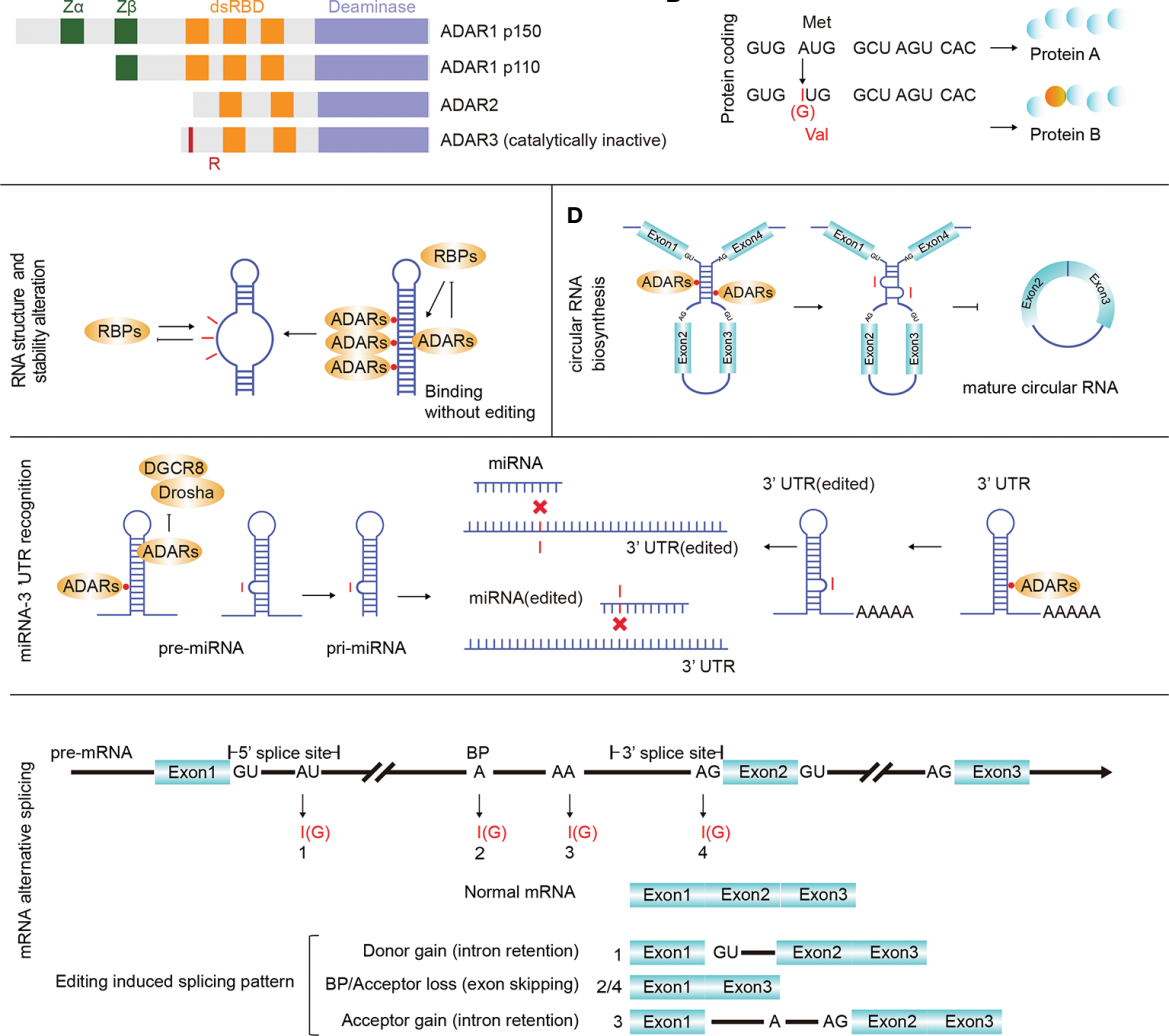
ADARs and RNA editing effects. **(A)** There are three main proteins of ADAR enzymes, ADAR1 (p110 and p150), ADAR2, and ADAR3. Vertebrate ADARs share a conserved deaminase domain and two to three dsRNA-binding domains (dsRBDs). In addition, ADAR1 p110 and p150 have similar Z-DNA-binding domains. ADAR3 is unique since its deaminase domain is catalytically inactive, and it also has an arginine-rich domain (R). **(B)** RNA editing in gene coding regions may introduce protein mutations. **(C)** Binding ADARs to certain dsRNAs may affect the RNA structure, thereby altering RNA biological processing and stability. **(D)** ADAR1 binds and inhibits the generation of circular RNAs. **(E)** microRNA (miRNA) or 3’ UTR editing may change or redirect the interactive relationship between certain UTRs and miRNAs. **(F)** RNA editing sites were identified in all three main regions involved with pre-mRNA alternative splicing (donor: 5’ splicing site, acceptor: 3’ splicing site and branch site), and pre-mRNA intron editing may contribute to pre-mRNA alternative splicing.

In mammals, ADAR-induced adenine to inosine (A-to-I) is a widespread primary type of RNA editing (1). As adenosine deaminases, ADAR proteins are able to bind to both intracellular and extranuclear double-stranded RNA (dsRNA), producing inosine (I) from adenosine (A) by deamination on RNA coding and non-coding regions. Since inosine prefers to pair with cytidine (C), researchers have also recognized A-to-I RNA editing as A-to-G (guanine) editing (2). ADAR proteins include three types in mammals, ADAR1, ADAR2 (ADARB1), and ADAR3 (ADARB2) (**Figure 1A**). ADAR1 and ADAR2 reside in most human tissues and are the major mediators of A-to-I RNA editing. Without deaminase activity, ADAR3 is mainly expressed in the brain. Recent research has indicated that ADAR3 may disturb ADAR2 function by acting as a competitive inhibitor (3). Moreover, ADAR1 has two isoforms resulting from the alternative promoters ADAR1 p110 and ADAR1 p150. ADAR1 p110 is constitutively expressed, while ADAR1 p150 is inducible by interferons (IFNs). When dsRNA sensors (such as MDA5 and PKR) in cells sense the presence of exogenous nucleic acids, they can induce the generation of IFNs and activate ADAR1 p150 (4).

Although past research has indicated that ADARs and A-to-I RNA editing are essential for multiple biological processes, abnormal expression or editing levels can trigger various diseases (5, 6). ADAR1 is required for mammalian early development (7–10), null ADAR1 expression causes embryonic death in mice (11, 12), and knocking out MDA5 can rescue the ADAR^−/−^ embryonic phenotype because MDA5 is responsible for distinguishing and helping remove exogenous dsRNA, except for ADAR-edited dsRNA (13). ADAR1 is a suppressor of interferon signaling (7) and controls innate immune responses to exogenous RNA (14). Abnormal expression of ADAR1 results in IFN production, which may take part in enhancing autoimmunity and inducing systemic lupus erythematosus to a certain degree (15). ADAR1&2 expression is positively correlated with the proliferative activity of most cells and inflammatory responses, especially playing a vital role in the occurrence and development of several cancers (16).

RNA editing affects many basic biological processes. When editing occurs in the mRNA coding region, it may cause mutations that increase the regulation diversity at both the transcriptional and proteomic levels (**Figure 1B**). When editing occurs in the non-coding RNA region, it can affect the RNA secondary structure (**Figure 1C**), circular RNA biosynthesis (**Figure 1D**), microRNA (miRNA)-mRNA targeting (**Figure 1E**) and mRNA alternative splicing (**Figure 1F**). Editing-induced RNA secondary structure alterations may affect the related protein abundance by changing the RNA stability (17–19). Therefore, accurate identification of RNA editing sites in cancer is important for investigating cancer development. Currently, many RNA editing identification bioinformatics strategies and software tools have been developed. Using these tools and algorithms, researchers have systematically identified RNA editing sites (20–22) on a large scale. At present, these identified human RNA editing sites are mainly summarized in four databases, REDIportal (http://srv00.recas.ba.infn.it/atlas/index.html) (21), DARNED (https://darned.ucc.ie/) (23), RADAR (http://rnaedit.com/) (24), and CLAIRE (http://srv00.recas.ba.infn.it/atlas/claire.html) (25). There are about 15.6 million editing sites in REDIportal, 0.2 million in DARNED, 2 million in RADAR, and 1,147 in CLAIRE, and REDIportal almost covered all human RNA editing sites of these four databases. Statistical analysis of these RNA editing site gene regions showed that most sites resided in non-coding RNA regions. In fact, protein-coding RNA regions account for only 2% of the human genome (26), while a large number of regions are non-coding regions, and most of the known RNAs are non-coding RNAs (ncRNAs). ncRNAs come from a wide range of sources and are abundantly expressed. While many rRNAs and tRNAs have high abundance, some ncRNAs, such as miRNAs, circular RNAs, long non-coding RNAs (lncRNAs), and Piwi-interacting RNAs (piRNAs), have low abundance (27). ncRNAs play a vital role in tumor regulation (28), and multiple ncRNAs interact in tumors, forming a competing endogenous RNA (ceRNA) network in cancer formation (29). Abnormal expression of some ncRNAs, such as miRNAs and lncRNAs, can affect cancer occurrence and progression. Moreover, the level of RNA editing in non-coding regions was identified to be significantly associated with cancer patient survival (30). As a special ring-shaped ncRNA, circular RNA is usually produced during the back-splicing of exons. Because of its circular structure property, it is more stable than linear RNA. Current studies have shown that circular RNAs mainly function by acting as miRNA sponges and interacting with RNA-binding proteins and lncRNAs. Circular RNA also plays an important role in multiple cancer types and can be used as a cancer biomarker (31). Due to its unique formation mechanism, the generation of circular RNA is easily affected by the expression of ADARs. Many studies have shown that ADARs can inhibit the synthesis of circular RNAs (32–34).

As a novel characteristic of tumors, certain genes display different expression patterns in cancer patients and show distinct RNA editing levels that vary within the same patient in different tissues. Interestingly, high RNA editing levels posttranscriptionally increase the heterogeneity of genes, including both oncogenes and tumor suppressors, in cancer. Researchers have previously investigated the RNA editing levels between tumor and paracarcinoma tissues from the TCGA database (35–38), including sites in coding regions and non-coding regions (miRNAs, intergenic regions, etc.) and have analyzed functional RNA editing events (16, 39, 40). Furthermore, ADARs were found to be highly expressed in Lgr5^+^ cells (controversial cancer stem cells) (41). In addition to DNA mutations, RNA editing caused by ADARs significantly increases the RNA abundance, which could increase the protein heterogeneity in tumor cells and induce tumor drug resistance (39). The identification of RNA editing sites in tumors will help us study the mechanism of tumorigenesis and identify some tumor-specific molecular markers. However, because of the high DNA mutation in tumors, it is a challenge to identify RNA editing sites accurately.

It is difficult to predict the function of RNA editing sites located in non-coding regions owing to their diversity and interactions with various ncRNAs. Moreover, except for typical RNA editing sites (such as those resulting in proteins and microRNA seed region alterations) that can be intuitively selected according to their locations, many other potentially functional RNA editing sites residing in non-coding regions still need to be explored and validated (42). This problem is complicated by the lack of effective judging and predicting tools.

This review outlines the existing bioinformatics strategies for identifying editing sites in tumors, which can be used for further experimental verification of downstream effects and clinical relevance. Simultaneously, we provide some suggestions for ncRNA editing research and the potential application of ADAR inhibitors in the treatment of cancer.

## RNA Editing Site Identification

In 1991, the first A-to-I RNA editing report was published when researchers detected RNA editing events on GluR mRNA (43). At the same time, four editing sites on 5-HT(2c) mRNA were identified (44). As DNA/RNA sequencing technology has developed, abundant high-seq data have made it possible to search RNA editing sites and analyze RNA editing levels by comparing RNA-seq data to related DNA-seq data, even using RNA-seq data alone (45–47). In addition, there are several experimental methods that directly detect inosine, including ICE-seq (48), EndoVIPER-seq (49), and other methods for capturing inosine (50–52). However, these methods also have obvious defects. (1) The effects of enzymatic or chemical treatments are usually incomplete, and RNase T1 will also induce RNA degradation. (2) RNA modifications could directly disturb reverse transcription (53), such as m1A (54, 55), and this effect induces many false-positive results that must be corrected with complex bioinformatics methods.

### Lateral Computational A-to-G RNA Editing Site Calling Strategy

Editing site calling is a complicated process involving many aspects and requiring appropriate optimization and high accuracy in each step. Therefore, researchers have summarized some functionally integrated pipelines (56–59). Currently, there are two popular analysis methods targeting A-to-I RNA editing, including comparing the RNA-seq data with its DNA-seq data and directly analyzing the RNA-seq data alone. In 2012, several groups separately reported their optimized RNA editing calling methods by directly comparing the RNA-seq data with its corresponding DNA-seq data (60–62). Since there is no need to delete SNPs (single-nucleotide polymorphisms), the editing identification accuracy will be improved in theory. At present, it is acknowledged that directly comparing RNA-seq data with the original DNA-seq data is the most ideal strategy by which to perform RNA editing calling. Unfortunately, matched DNA and RNA sequencing data from the same sample are not always available. To decrease costs and reduce processing times, researchers prefer to adopt RNA-seq alone to search for editing events. Until 2013, detection methods using tissue- and cell-specific RNA-seq alone were reported by several different groups (46, 63). This strategy has substantially promoted research in related fields, and it is thought to be useful in other organisms carrying suitable reference genomes.

As mentioned above, the two popular strategies share a common calling strategy that includes the following four steps: (1) preprocessing sequencing data, (2) sequencing read mapping, (3) RNA editing calling, and (4) RNA editing site annotation. When analyzing RNA editing levels, studies usually employ certain common mapping tools, such as BWA (64), Bowtie (65), HISAT2 (66), GSNAP (67), and STAR (68). Interestingly, the same high-seq data usually display low repeatability when processed by different mapping tools; therefore, some specific tools for RNA editing calling have been developed, such as RASER (69). However, recent integrated pipelines for RNA editing analysis usually require specific mapping tools according to each developer’s optimization. RNA editing calling is the key step that identifies true RNA editing events according to DNA-RNA mismatches. A conventional identification method is to identify DNA/RNA mismatches in samples using the tools HaplotypeCaller in GATK, Samtools (70), VarScan 2 (71), etc., followed by removing SNPs and DNA mutations. Researchers have also developed a large number of tools for accurate identification of RNA editing. For instance, to filter out these false-positive events, REDItools provided a threshold according to an empirically observed distribution (47). We summarize the popular RNA editing bioinformatics tools in **Table 1**.

**Table 1:** Main bioinformatics tools for RNA editing detection.

Tools	Required sequencing data	URL	Ref
REDItools	RNA-seq or RNA and DNA-seq	https://github.com/BioinfoUNIBA/REDItools	(47)
RES-Scanner	RNA-seq and DNA-seq	https://github.com/ZhangLabSZ/RES-Scanner	(72)
JACUSA	RNA-seq or RNA and DNA-seq	https://github.com/dieterich-lab/JACUSA	(73)
GIREMI	RNA-seq	https://github.com/zhqingit/giremi	(45)
RNAEditor	RNA-seq	http://rnaeditor.uni-frankfurt.de/	(74)
DeepRed	RNA-seq	https://github.com/wenjiegroup/DeepRed	(75)
RED-ML	RNA-seq or RNA and DNA-seq	https://github.com/BGIRED/RED-ML	(76)
SPRINT	RNA-seq	https://sprint.tianlab.cn/	(77)
RDDpred	RNA-seq	http://epigenomics.snu.ac.kr/RDDpred/	(78)
Rcare	RNA-seq or RNA and DNA-seq	http://www.snubi.org/software/rcare/	(79)
DREAM	miRNA-seq	http://www.cs.tau.ac.il/~mirnaed/	(80)
RASER	RNA-seq	https://www.ibp.ucla.edu/research/xiao/RASER.html	(69)
InosinePredict	RNA-seq	http://hci-bio-app.hci.utah.edu:8081/Bass/InosinePredict	(81)
VIRGO	RNA-seq	https://github.com/InfOmics/VIRGO	(82)
AIRLINER	RNA-seq	http://alpha.dmi.unict.it/airliner/	(83)
PAI	RNA-seq	N/A	(84)
iRNA-AI	RNA-seq	N/A	(85)
EPAI-NC	RNA-seq	N/A	(86)
isoTar	RNA-seq	https://ncrnaome.osumc.edu/isotar/	(87)
REP	RNA-seq or RNA and DNA-seq	http://www.rnaeditplus.net/	(88)
RED	RNA-seq or RNA and DNA-seq	https://github.com/REDetector/RED	(89)
PRESa2i	RNA sequences	http://brl.uiu.ac.bd/presa2i/index.php	(90)

### Current Situation and Challenge of RNA Editing Calling

Sequencing errors, DNA mutations, and a lack of a suitable SNP database will result in false-positive results that affect RNA editing detection. These sequencing errors are mainly caused by reverse transcription, homopolymers, low-quality sequences, etc. An important factor resulting in sequencing errors is RNA modification, such as m1A and m6A. The reverse transcriptase will likely misidentify modified nucleotides as other types of nucleotides (91) [e.g., m1A is usually recognized as G instead of A (92)], which will produce many false-positive editing sites in the final results. In addition, tumor tissues are usually stored in paraffin or formalin for further research, and chemical reagents may damage DNA and RNA in these samples and ultimately affect the quality of the DNA/RNA library. To acquire high-quality sequencing reads, researchers usually perform several corrective processes. Pinto et al. summarized the related progress and provided several necessary remarks in their review (57). The processes mainly included adjusting the read quality (QC) threshold value to over 20, removing low-quality reads and using the random sequencing primer adaptor.

Upon removing SNPs, several tools, such as REDItools (47) and RNAEditor (74), automatically compare the data with the SNP databases. Researchers have established several SNP datasets, including dbSNP and HapMap (93). Plainly, the SNP database quality and selection are important determinants for analyzing the editing level using RNA-seq alone. Interestingly, it appears that a problem is caused by the accuracy of the dbSNP database remaining uncertain, since some SNP cases reported in past experimental results have recently been reclassified as RNA editing (94). For this reason, researchers have developed many other tools that remove SNPs better. GIREMI includes a mutual information (MI) model that is able to directly remove SNPs without comparing the data to a reference SNP database (45), and SPRINT is capable of directly identifying RNA editing events *via* a novel SNP-free algorithm (77).

### Improvements in Editing Site Calling for Cancer Research

**Figure 2:**
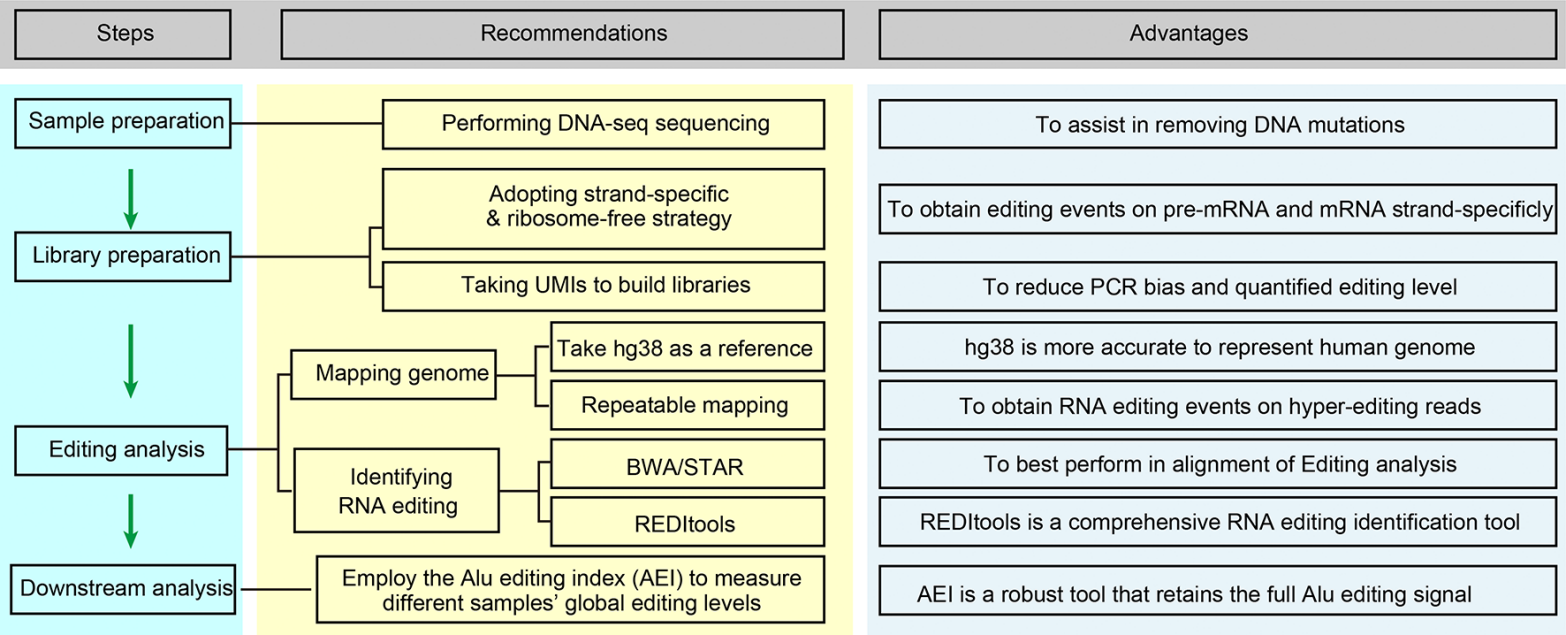
Optimized editing sites identification strategies for cancer research. This flow chart is briefly regarding the content of *Improvements in Editing Site Calling for Cancer Research*.

Although the detection of editing sites with high-seq data is widely accepted and utilized, systematic optimization for accurately measuring RNA editing is still insufficient. Here, we offer several suggestions for applications using next-generation RNA sequencing for cancer RNA editing research (**Figure 2**).

1. Perform DNA-seq sequencing of the same sample if available. Tumor tissues generally have a high DNA mutation rate, and the filtration of DNA mutations is very difficult without DNA-seq sequencing. The method commonly used is to refer to previously reported DNA mutation data, such as the COSMIC database (35, 95).
2. Adapt the strand-specific and ribosome-free strategy for preparing the RNA-seq library. This will improve the accuracy of editing calling, yield more editing events on unspliced pre-mRNA fragments, and obtain more information from non-coding RNAs. Generally, the abundance of some ncRNAs, such as circular RNAs, is low, so we can obtain more circular RNAs or improve the depth of sequencing by removing linear RNAs through RNase R treatment.
3. Using hg38 as a reference genome and repeatable mapping is feasible for improving the fault-tolerant ability for hyperediting reads. Recent research indicates that the RNA editing site location usually displays a clustering pattern (96), and sequencing reads containing multiple mismatches are considered to be hyperediting. Porath et al. developed a specific method that recognized all A as G in unmapped reads before the mapping process to avoid the excessive deletion of hyperediting reads (97). Subsequently, Picardi et al. analyzed human hyperediting levels from different tissues *via* this method (22). Therefore, we recommend referring to Porath’s strategy to analyze hyperediting reads.
4. Employ the Alu editing index (AEI) to measure the global editing levels in different samples. Erez Y. Levanon and Eli Eisenberg et al. provided the Alu editing index (AEI) to measure the global editing levels in different samples. The AEI ratio weighted by A-to-G mismatches within Alu repeats relative to the total number of adenosines within Alu elements represents the average Alu editing levels, indirectly showing the overall RNA editing levels (36, 98).
5. For the identification of RNA editing in tumor samples with both DNA-seq and RNA-seq data, we suggest BWA (DNA and RNA-seq) or BWA (DNA-seq) + STAR (RNA-seq) for mapping the sequencing data and REDITools for RNA editing calling. Maria et al. compared some commonly used alignment tools, including BWA, GSNAP, HISAT, and STAR, and RNA identification tools, such as RNAEditor, GIREMI, REDItools, RES, and JACUSA, and analyzed the ability of these tools to identify RNA editing (59). In their findings, BWA and STAR achieved the best alignment. REDItools is a more comprehensive RNA editing identification tool with high accuracy that can analyze the data obtained from various strategies of library construction (stranded or non-stranded RNA-seq) and provide additional options, allowing researchers to filter (SNP or DNA mutations) and annotate (genomic region or Alu region) the editing sites with their own files. To our delight, Picardi shares their updated protocol, which is a relatively systematic and detailed RNA editing identification process, for identifying RNA editing sites (99). The protocol explains how to analyze the original DNA/RNA sequencing data and obtain the candidate RNA editing sites in detail. Taking Huntington disease (HD) as an example, it also introduces how to compare the differences in RNA editing levels in different tissues. Moreover, they also developed high-performance HPC-REDItools for large-scale samples, which greatly improves the speed of operation (100). For the identification of RNA editing in tumor samples with only RNA-seq, we recommend HISAT2 to handle RNA-seq data and REDITools to conduct RNA editing calling. We compared several RNA editing identification processes using YH’s RNA-seq data and concluded that this combination is better in terms of speed and accuracy (88). At present, some researchers only focus on the known editing sites in existing RNA editing databases, such as RADAR and REDIportal. In our opinion, especially for cancer RNA editing research, *de novo* identification of these unknown RNA editing sites is necessary.
6. We suggest selecting unique molecular identifiers (UMIs) when building RNA-seq libraries, which will bring many advantages (101): PCR mutations will be directly removed, the editing levels will be absolutely quantified according to the accurate number of edited RNAs, and the computational operational process will be simplified. However, this novel method requires further refined algorithms and processing flows.

## The Effects of ADARs Induced A-to-I RNA Editing in Cancer

Based on tens of thousands of potential RNA editing sites reported from bioinformatics methods, many experimentally validated A-to-I editing sites and their regulatory mechanisms have been demonstrated. As shown in **Figure 1B**, editing in mRNA may results in missense mutations and alterations in the beginning and terminating translation (102). Multiple editing sites located in certain coding regions, such as AZIN1 (103, 104), GABRA3 (105, 106), and COPA (40, 107, 108), have been shown to affect tumor progression. According to the reported databases, most editing sites reside within non-coding regions (>90%), and RNA editing has been detected in many types of ncRNAs, including piRNAs (109). Here, we briefly summarize the mechanisms of several typical editing effects in non-coding regions. (1) Editing occurs in the 5’ splice site, branch point, and 3’ splice site and is able to affect pre-mRNA alternative splicing. (2) When combined with pre-miRNA or pri-miRNA, ADARs inhibit Drosha and Dicer1 functions, affecting miRNA maturation and expression. The editing-induced sequence changes in mature miRNAs (especially in the seed sequence) or in the 3’ UTR can disturb their specific interactions. (3) For long non-coding RNAs, several reports analyzing editing levels have been published (110–112), and partial editing sites having direct effects have been reported (113). (4) Since circular RNAs are byproducts of RNA splicing, editing effects on RNA splicing theoretically affect circular RNA expression. Researchers have observed high levels of A-to-I editing in circular RNA precursors and have confirmed that ADAR is related to its formation (32, 33). (5) Editing sites occurring in the 3’ UTR or intron region are able to affect the RNA structure and stability (17–19). For convenience, we list recently reported tumor-effectible RNA editing sites in **Supplementary Table S1**.

### RNA Editing Events in Non-Coding Regions Are Involved With Cancer

There are a large number of RNA editing phenomena in ncRNAs (114), and researchers have identified many RNA editing sites located in ncRNAs, such as lncRNAs (112). In addition, several specific databases aimed at ncRNAs have been built, including MiREDiBase (https://ncrnaome.osumc.edu/miredibase/) (for miRNA) (115) and LNCediting (http://bioinfo.life.hust.edu.cn/LNCediting/) (for lncRNA) (110). Several typical editing effects that occur on ncRNAs are listed below. (1) ADAR1-mediated miR-200 overediting affects an oncogene in thyroid cancer (116). The overediting of miR-200 weakens its interaction with and targeting of ZEB1, resulting in inhibition of epithelial-mesenchymal transition (EMT). (2) In prostate cancer, ADAR1 promotes cell proliferation by editing lncRNAs and PCA3 and improving the stability and expression of PCA3, thereby inhibiting the tumor suppressor PRUNE2 (113). (3) In glioma tumors, ADAR2 inhibits cell migration and invasion by editing miR-376a-1 and shifting the targeted gene from RAP2A to AMFR (117). (4) In melanoma, ADAR1 attenuates the inhibition of CPEB1 by miR-455-5p by editing miR-455-5p, which promotes the proliferation and metastasis of melanoma (118). (5) Zipeto et al. found that the edited miRNA Let-7 is a main factor promoting leukemia cell self-renewal (119). (6) Hepatocellular carcinoma (HCC) and the android receptor (AR) promote the expression of ADAR1 p110, while ADAR1 p110 inhibits circular RNA (hsa_circ_0085154) expression and finally inhibits the proliferation of HCC tumors (120).

### Search Strategies for Effective RNA Editing Events in Cancer

Tumor and adjacent tissue samples are suitable subjects for studying RNA editing—a large number of non-cancer-specific RNA editing sites can be excluded by comparing RNA editing in cancer and adjacent tissue. The investigation range of essential RNA editing sites affecting cancer occurrence and development can be effectively narrowed by comparing the editing levels in cancer and adjacent tissue. Most cancers are accompanied by detailed clinical data, from which the characteristics of RNA editing in different cancers can be summarized and the range of key targets can be further streamlined.

ADAR1&2 expression is different in various cancers. ADAR1 expression increases in most cancer types, such as breast invasive carcinoma and liver hepatocellular carcinoma, but decreases in a few cancers, such as kidney chromophobe. Some researchers found that the expression of ADAR1 and ADAR2 in the same cancer can be totally opposite. For example, some studies show that ADAR1 is a potential tumor enhancer with high levels, and ADAR2 is recognized as a tumor suppressor in HCC (40, 108).

The role of specific RNA editing level changes mediated by different ADAR enzymes in tumors has been partially discovered. To further determine which RNA editing site is closely related to tumor development and maintenance, researchers can select paired samples from cancer patients and identify these tumor-related RNA editing sites using statistical methods such as Fisher’s exact test and Wilcoxon rank sum and signed rank test. Studies have shown that certain site editing levels are greatly associated with patient survival (30). Therefore, we can link the editing level of RNA editing sites with clinical data such as the tumor clinical stage, cancer subtype and patient survival. In addition, Han et al. showed that editing levels of certain genes are associated with tumor drug sensitivity, which could be used as a potential screening strategy in clinical medication (35).

Based on the initial selection strategies mentioned above, a large number of candidate editing sites related to cancer could be identified, and we can further narrow the range according to previously reported important tumor-related genes. Additionally, since the level of RNA editing is regulated by ADAR1 and/or ADAR2, we can determine which enzyme acts on a specific editing site by analyzing the correlation between the expression levels of ADAR1/2 and editing sites. Moreover, RNA editing affects the gene expression level in various ways, so we were able to analyze whether there was a correlation between the change in the editing level of RNA editing sites and the expression level of the gene in which RNA editing sites were located. Finally, a necessary confirmation step should be performed for the newly identified editing sites *via* Sanger sequencing (121), mmPCR-seq (122), or RESSq-PCR (123).

### Predicting the Function of RNA Editing Events in Cancer

Predicting the capabilities of editing is challenging. Before carrying out experiments to verify the abovementioned tumor-related editing sites, we can roughly analyze the impact of these RNA editing sites by *in silico* prediction to guide subsequent experiments. RNA editing sites are distributed in different regions of the gene, such as CDS, introns, and UTRs, so it is vital to distinguish different locations of these editing sites when predicting their functions. RNA editing sites occurring in CDS may cause non-synonymous amino acids or lead to early termination of mRNAs, and many tools, such as ANNOVAR (124), VEP (125), SnpEff (126), and SnpSift, can predict these sites. Julie et al. compared the three tools and discovered that VEP and SnpEff can better annotate the mutations of different transcripts, which is helpful for the functional prediction of RNA editing sites (127). In fact, 95% of nascent pre-mRNA could be influenced by RNA editing (128). Some predictable RNA editing sites are involved in splicing, and the conventional way of detecting these sites is predicting the conserved 5’ splice site and 3’ splice site and then analyzing the proportion between variant RNAs after editing occurs and the original RNAs, which is also called the percent spliced in index (PSI) (129). For RNA editing sites that occur in 3’ UTR, a large number of current studies have revealed miRNA interactions, and there are also multiple tools that can analyze the relationship between mutant RNAs and the corresponding miRNAs (130). In addition, a few RNA editing events lead to alterations of the RNA structure, and these editing cases that change the free energy of RNAs can be predicted by platforms such as RNAfold (131) and STRUM (132).

To predict functional editing sites with high efficiency, we took the lead in developing an *in silico* online analysis system, RNA Editing Plus (REP), that effectively calls and annotates human A-to-I RNA editing events, predicting their downstream effects on pre-mRNA alternative splicing and miRNA-3’ UTR targeting *via* human high-seq data (88). We believe that our platform governing multiple optimized prediction methods will assist more scientific groups in investigating their targets of interest in cancer.

### Effects of ADARs Induced A-to-I Editing on Circular RNAs

ADARs can significantly affect the biosynthesis of circular RNAs (32–34). We summarize that ADARs affect circular RNAs in two ways. In the first effect, despite the lack of direct evidence, theoretically, RNA editing sites located in the recognition region of 5’ splice site and 3’ splice site could directly affect the generation of circular RNAs. When RNA editing takes place in the 5’ splice site and 3’ splice site regions, it not only affects pre-mRNA splicing but also further changes the splicing mode of mRNA, which may directly affect the generation of circular RNAs. In addition, it has been pointed out that approximately 99.2% of circular RNAs require 5’ splice sites and 3’ splice sites simultaneously (133), so if RNA editing occurs in these regions, most of the circular RNAs will be directly affected. On the other hand, the formation of dsRNA structures is accompanied by the formation of circular RNAs. Since ADARs can act on these regions and produce A-to-I RNA editing, the structure of dsRNA is destroyed, and the biosynthesis of circular RNAs is affected. In conclusion, changes in ADAR1 expression could directly influence the biosynthesis of circular RNAs.

Interestingly, mutations resulting from RNA editing occurring in pre-mRNA could be transmitted into mature circular RNAs. For instance, Hosaka et al. reported a circular RNA edited by ADAR2 named circGRIA2 (hsa_circ_0125620) in mouse spinal motor neurons and human SH-SY5Y cells, and circGRIA2 editing level alteration is a potential marker for early serum diagnosis of amyotrophic lateral sclerosis (ALS) since it can be secreted out of the cell (134). They claimed that circular RNA can be used as a marker for the early diagnosis of neoplastic diseases because it can be excluded from extracellular properties. However, the clinical feasibility of blood tests for measuring circular RNAs needs to be further validated. As mentioned earlier, in cancer, various types of ncRNAs, such as circular RNAs, miRNAs, mRNAs, and lncRNAs, can work together to form a ceRNA regulatory network. miRNA plays a vital role in this process. As sponges of miRNAs, circular RNAs can inhibit the function of miRNAs. In addition, we can use the aforementioned splicing prediction tools to analyze the changes in RNA editing at 5’ splice site and 3’ splice site through RNA-seq sequencing data (128), thus directly predicting the changes in circular RNAs.

### Application of ADARs Induced A-to-I Editing in Cancer Therapy

Since the ability of ADARs to deaminate has also been applied to the field of gene editing, they also have great potential in cancer therapy (135, 136). Nevertheless, recent findings have pointed us toward new avenues to identify the posttranscriptional regulatory mechanisms in cancer research. The editing of endogenous dsRNA by ADARs was found to be required to prevent innate immune system activation (14, 137, 138). Two groups also showed that knockdown of ADAR1 reduced the sensitivity of several tumor cells to antitumor drugs by activating interferons (IFNs), meaning that ADAR1 is able to enhance the effects of certain tumor immune drugs (139, 140). In addition, it has been shown that RNA editing is associated with drug resistance in tumors, and some clinically relevant RNA editing sites occurring on ncRNAs have also been demonstrated (30). Overall, ADAR1 and RNA editing can be used as targets in cancer immunotherapy (141) to treat cancer together with tumor immune drugs.

It has been found that some chemically synthesized small-molecule drugs can inhibit the expression of ADAR1. Ding et al. reported that 8-chloro-adenosine can inhibit the ADAR1/p53 pathway, inhibiting the proliferation of breast cancer (142). Erythro-9-(2-hydroxy-3-nonyl) adenine hydrochloride (EHNA) has also been proven to be an inhibitor of ADARs (143). Targeted inhibitors are considered to be effective in cancer treatment, and these small-molecular drugs are currently mainly divided into artificial drugs and natural products. There are some effective components in natural drugs that can inhibit cancers and have been used in clinical treatments. For example, paclitaxel extracted from plants is an effective antitumor drug (144). This is also why some researchers have used a variety of methods to identify the ingredients of important natural products to treat cancer (145). However, there are few studies on the active components of natural products as inhibitors of ADARs. Natural compounds may change the level of ADAR-mediated editing in tumors or play an anticancer role by virtue of the non-editing function of ADARs, which will provide a new research direction for the potential of ADARs in cancer therapy.

## Conclusions and Future Perspectives

To date, great efforts have been made to develop computational methods alongside the advancement of sequencing technologies to detect RNA editing events, and millions of editing sites have been reported, allowing researchers to gain growing information on different tissues. However, many aspects could be optimized to explore tissue-specific editing levels in the future. In addition, the advent of third-generation long-read sequencing technologies such as Pacific Biosciences and Oxford Nanopore brings about more facilities for editing calling since it will theoretically circumvent the current technical bottlenecks, such as PCR errors and hyperediting read loss (96, 146, 147). On the other hand, more high-seq data from single cells have been made public, providing necessary information for unraveling RNA editing effects on cell diversity at the single-cell level. Interpreting the level of RNA editing at the single-cell level in cancer has a great promoting effect on our further understanding of tumor heterogeneity and the development of tumor heterogeneity. Although initial research has been reported on the human brain (148), reads with low abundance and coverage have restricted the application of these data (57). As mentioned earlier, RNA editing located in the non-coding region is most abundant in cancer. It is also urgent to clarify the functions of RNA editing sites and apply them to the treatment of tumors. RNA editing could dramatically increase ncRNA abundance, while ncRNAs such as lncRNAs are able to affect the drug resistance of tumors (149). Therefore, in-depth mining of the mechanism of RNA editing in lncRNAs facilitates our in-depth understanding of tumor heterogeneity, helping us treat cancer. There is a link between RNA editing and drug sensitivity—for example, the levels of RNA editing of COG3 and COPA have a strong correlation with drug sensitivity (37), which indicates that RNA editing has great potential in cancer therapy and drug development.

## Author Contributions

Conceptualization: YZ and GS. Writing—original draft preparation: YZ, HW, and SC. Writing—review and editing: all authors. Visualization and table collection: HW and JW. Funding acquisition: YZ and GS. All authors contributed to the article and approved the submitted version.

## Funding

This research was funded by National Natural Science Foundation of China, grant number 31502030 to YZ and grant number 81700550 to GS.

## Conflict of Interest

The authors declare that the research was conducted in the absence of any commercial or financial relationships that could be construed as a potential conflict of interest.
