# Supplementary material for: A-to-I RNA Editing in Cancer: From Evaluating the Editing Level to Exploring the Editing Effects
Source: Front Oncol. 2021 Feb 11;10:632187. doi: 10.3389/fonc.2020.632187 (PMC7905090; doi:10.3389/fonc.2020.632187)
Supplement: Supplementary file 1 [file Table_1.docx]

Supplementary Material

**Supplementary Table S1. Data Validated functional A-to-I RNA editing sites in human.**

| Chromosome | Position  (hg38) | Gene | Effects | ADAR  responsible | Ref |
| --- | --- | --- | --- | --- | --- |
| Chr1 | 160332454 | COPA | Protein mutation  (I>V) | ADAR2 | (1-3) |
| Chr1 | 1167164 | hsa-mir-200b | Redirection of miRNA binding | ADAR2 | (4-5) |
| Chr2 | 46576601 | RHOQ | Protein mutation (N>S) | N/A | (6) |
| Chr2 | 38566188 | HNRPLL | Changing splicing pattern | ADAR1&2 | (7) |
| Chr2 | 38566185 | HNRPLL | Changing splicing pattern | ADAR1&2 | (7) |
| Chr2 | 38566176 | HNRPLL | Changing splicing pattern | ADAR1&2 | (7) |
| Chr3 | 37969477 | hsa-mir-26a-1 | Affecting miRNA processing | ADAR1 | (8) |
| Chr3 | 58156064 | FLNB | Protein mutation (M>V) | ADAR 1&2 | (2) |
| Chr3 | 58156074 | FLNB | Protein mutation (Q>R) | N/A | (3) |
| Chr4 | 57110068 | IGFBP7 | Protein mutation (K>R) | ADAR2 | (9) |
| Chr4 | 57110120 | IGFBP7 | Protein mutation (R>G) | N/A | (3) |
| Chr4 | 77058527 | CCNI | Protein mutation (R>G) | ADAR1 | (1) |
| Chr5 | 143225274 | ARHGAP26 | Redirection of 3’ UTR binding | ADAR1 | (10) |
| Chr5 | 143225281 | ARHGAP26 | Redirection of 3’ UTR binding | ADAR1 | (10) |
| Chr5 | 143225441 | ARHGAP26 | Redirection of 3’ UTR binding | ADAR1 | (10) |
| Chr5 | 149732884 | hsa-mir-378a | Redirection of miRNA binding | ADAR1 | (11) |
| Chr5 | 157309800 | CYFIP2 | Protein mutation (K>E) | ADAR2 | (12) |
| Chr6 | 2948933 | SERPINB6 | Protein mutation (E>G) | N/A | (3) |
| Chr6 | 44252185 | HSP90AB1 | Protein mutation (K>R) | N/A | (3) |
| Chr6 | 73519351 | EEF1A1 | Protein mutation (T>A) | N/A | (3) |
| Chr6 | 73519393 | EEF1A1 | Protein mutation  (I>V) | N/A | (3) |
| Chr6 | 160348633 | SLC22A3 | Protein mutation (N>D) | ADAR2 | (13) |
| Chr7 | 5495852 | hsa-mir-589 | Redirection of miRNA binding | ADAR2 | (14) |
| Chr7 | 17345106 | AHR | Redirection of 3’ UTR binding | ADAR1 | (15) |
| Chr7 | 39950745 | CDK13 | Protein mutation (Q>R) | N/A | (1) |
| Chr7 | 131510316 | PODXL | Protein mutation (H>R) | ADAR2 | (16) |
| Chr8 | 102829408 | AZIN1 | Protein mutation (S>G) | ADAR1 | (17-18) |
| Chr8 | 140692191 | PTK2 (FAK) | Impacting the RNA stability | ADAR1 | (19) |
| Chr9 | 94178843 | hsa-let-7d | Affecting miRNA processing | ADAR1 | (20) |
| Chr9 | 94178897 | hsa-let-7d | Affecting miRNA processing | ADAR1 | (20) |
| Chr9 | 96538502 | CDC14B | Impacting the RNA stability | ADAR2 | (21) |
| Chr9 | 96538515 | CDC14B | Impacting the RNA stability | ADAR2 | (21) |
| Chr9 | 96538523 | CDC14B | Impacting the RNA stability | ADAR2 | (21) |
| Chr9 | 96538550 | CDC14B | Impacting the RNA stability | ADAR2 | (21) |
| Chr9 | 96538552 | CDC14B | Impacting the RNA stability | ADAR2 | (21) |
| Chr9 | 96538554 | CDC14B | Impacting the RNA stability | ADAR2 | (21) |
| Chr9 | 96538593 | CDC14B | Impacting the RNA stability | ADAR2 | (21) |
| Chr9 | 96538601 | CDC14B | Impacting the RNA stability | ADAR2 | (21) |
| Chr9 | 96538613 | CDC14B | Impacting the RNA stability | ADAR2 | (21) |
| Chr9 | 96538616 | CDC14B | Impacting the RNA stability | ADAR2 | (21) |
| Chr9 | 96538617 | CDC14B | Impacting the RNA stability | ADAR2 | (21) |
| Chr9 | 96538625 | CDC14B | Impacting the RNA stability | ADAR2 | (21) |
| Chr9 | 96538641 | CDC14B | Impacting the RNA stability | ADAR2 | (21) |
| Chr9 | 114209450 | hsa-mir-455 | Redirection of miRNA binding | ADAR1 | (22) |
| Chr9 | 114209450 | hsa-mir-455 | Affecting miRNA processing | ADAR1 | (22) |
| Chr9 | 114209465 | hsa-mir-455 | Affecting miRNA processing | ADAR1 | (22) |
| Chr9 | 95085510 | hsa-mir-27a | Affecting miRNA processing | ADAR1 | (23-25) |
| Chr9 | 76785919 | PCA3 | Affecting lncRNA regulation | ADAR1 | (26) |
| Chr9 | 76785950 | PCA3 | Affecting lncRNA regulation | ADAR1 | (26) |
| Chr10 | 80627574 | DHFR | Redirection of 3’ UTR binding | ADAR1 | (13) |
| Chr10 | 80627583 | DHFR | Redirection of 3’ UTR binding | ADAR1 | (13) |
| Chr10 | 80627604 | DHFR | Redirection of 3’ UTR binding | ADAR1 | (13) |
| Chr10 | 80627605 | DHFR | Redirection of 3’ UTR binding | ADAR1 | (13) |
| Chr10 | 80627611 | DHFR | Redirection of 3’ UTR binding | ADAR1 | (13) |
| Chr10 | 80628024 | DHFR | Redirection of 3’ UTR binding | ADAR1 | (13) |
| Chr10 | 80628036 | DHFR | Redirection of 3’ UTR binding | ADAR1 | (13) |
| Chr10 | 80628041 | DHFR | Redirection of 3’ UTR binding | ADAR1 | (13) |
| Chr10 | 80628047 | DHFR | Redirection of 3’ UTR binding | ADAR1 | (13) |
| Chr10 | 80628087 | DHFR | Redirection of 3’ UTR binding | ADAR1 | (13) |
| Chr10 | 80628100 | DHFR | Redirection of 3’ UTR binding | ADAR1 | (13) |
| Chr10 | 80628171 | DHFR | Redirection of 3’ UTR binding | ADAR1 | (13) |
| Chr10 | 80628179 | DHFR | Redirection of 3’ UTR binding | ADAR1 | (13) |
| Chr12 | 4912576 | KCNA1 | Protein mutation  (I>V) | ADAR2 | (27) |
| Chr12 | 6951955 | PTPN6 | Changing splicing pattern | N/A | (28) |
| Chr12 | 57470841 | GLI1 | Protein mutation (R>G) | ADAR 1&2 | (29) |
| Chr12 | 68843224 | MDM2 | Redirection of 3’ UTR binding | N/A | (30) |
| Chr12 | 68843227 | MDM2 | Redirection of 3’ UTR binding | N/A | (30) |
| Chr12 | 68843230 | MDM2 | Redirection of 3’ UTR binding | N/A | (30) |
| Chr12 | 68843772 | MDM2 | Redirection of 3’ UTR binding | ADAR1 | (8) |
| Chr13 | 45516236 | COG3 | Protein mutation  (I>V) | N/A | (3) |
| Chr14 | 101040790 | hsa-mir-376a-1 | Redirection of miRNA binding | ADAR2 | (31) |
| Chr14 | 101045940 | hsa-mir-381 | Redirection of miRNA binding | ADAR1 | (32-33) |
| Chr14 | 101045943 | hsa-mir-381 | Redirection of miRNA binding | ADAR1 | (32-33) |
| Chr14 | 101022075 | has-mir-379 | Redirection of miRNA binding | ADAR2 | (34) |
| Chr15 | 75353745 | NEIL1 | Protein mutation (K>R) | ADAR1 | (35) |
| Chr17 | 42318026 | STAT3 | Changing splicing pattern | ADAR1 | (36) |
| Chr17 | 42318064 | STAT3 | Changing splicing pattern | ADAR1 | (36) |
| Chr17 | 42318069 | STAT3 | Changing splicing pattern | ADAR1 | (36) |
| Chr17 | 42318091 | STAT3 | Changing splicing pattern | ADAR1 | (36) |
| Chr17 | 59841288 | hsa-mir-21 | Affecting miRNA processing | ADAR2 | (37) |
| Chr17 | 59841318 | hsa-mir-21 | Affecting miRNA processing | ADAR2 | (37) |
| Chr17 | 59841323 | hsa-mir-21 | Affecting miRNA processing | ADAR2 | (37) |
| Chr18 | 58451081 | has-mir-122 | Redirection of miRNA binding | ADAR2 | (38) |
| Chr19 | 18177741 | IFI30 | Protein mutation (T>A) | N/A | (3) |
| Chr20 | 37519131 | BLCAP | Protein mutation (K>R) | ADAR 1&2 | (12; 39) |
| Chr20 | 37519161 | BLCAP | Protein mutation (Q>R) | ADAR 1&2 | (12; 39) |
| Chr20 | 37519170 | BLCAP | Protein mutation (Y>C) | ADAR 1&2 | (12; 39) |
| ChrX | 45746179 | hsa-mir-221 | Affecting miRNA processing | ADAR2 | (37) |
| ChrX | 45746242 | hsa-mir-221 | Affecting miRNA processing | ADAR2 | (37) |
| ChrX | 45746243 | hsa-mir-221 | Affecting miRNA processing | ADAR2 | (37) |
| ChrX | 45747042 | hsa-mir-222 | Affecting miRNA processing | ADAR2 | (37) |
| ChrX | 45747115 | hsa-mir-222 | Affecting miRNA processing | ADAR2 | (37) |

**Reference**

1. M. Zhang, J. Fritsche, J. Roszik, L.J. Williams, X. Peng, Y. Chiu, et al., RNA editing derived epitopes function as cancer antigens to elicit immune responses. Nat Commun 9 (2018) 3919. doi: 10.1038/s41467-018-06405-9

2. T.H. Chan, C.H. Lin, L. Qi, J. Fei, Y. Li, K.J. Yong, et al., A disrupted RNA editing balance mediated by ADARs (Adenosine DeAminases that act on RNA) in human hepatocellular carcinoma. Gut 63 (2014) 832-43. doi: 10.1136/gutjnl-2012-304037

3. X. Peng, X. Xu, Y. Wang, D.H. Hawke, S. Yu, L. Han, et al., A-to-I RNA Editing Contributes to Proteomic Diversity in Cancer. Cancer Cell 33 (2018) 817-828 e7. doi: 10.1016/j.ccell.2018.03.026

4. Y. Wang, X. Xu, S. Yu, K.J. Jeong, Z. Zhou, L. Han, et al., Systematic characterization of A-to-I RNA editing hotspots in microRNAs across human cancers. Genome Res 27 (2017) 1112-1125. doi: 10.1101/gr.219741.116

5. P.M. Shelton, A. Duran, Y. Nakanishi, M. Reina-Campos, H. Kasashima, V. Llado, et al., The Secretion of miR-200s by a PKCzeta/ADAR2 Signaling Axis Promotes Liver Metastasis in Colorectal Cancer. Cell Rep 23 (2018) 1178-1191. doi: 10.1016/j.celrep.2018.03.118

6. S.W. Han, H.P. Kim, J.Y. Shin, E.G. Jeong, W.C. Lee, K.Y. Kim, et al., RNA editing in RHOQ promotes invasion potential in colorectal cancer. J Exp Med 211 (2014) 613-21. doi: 10.1084/jem.20132209

7. Y.T. Chen, I.Y. Chang, H. Liu, C.P. Ma, Y.P. Kuo, C.T. Shih, et al., Tumor-associated intronic editing of HNRPLL generates a novel splicing variant linked to cell proliferation. J Biol Chem 293 (2018) 10158-10171. doi: 10.1074/jbc.RA117.001197

8. Q. Jiang, J. Isquith, M.A. Zipeto, R.H. Diep, J. Pham, N. Delos Santos, et al., Hyper-Editing of Cell-Cycle Regulatory and Tumor Suppressor RNA Promotes Malignant Progenitor Propagation. Cancer Cell 35 (2019) 81-94.e7. doi: 10.1016/j.ccell.2018.11.017

9. Y.B. Chen, X.Y. Liao, J.B. Zhang, F. Wang, H.D. Qin, L. Zhang, et al., ADAR2 functions as a tumor suppressor via editing IGFBP7 in esophageal squamous cell carcinoma. Int J Oncol 50 (2017) 622-630. doi: 10.3892/ijo.2016.3823

10. Q. Wang, H. Hui, Z. Guo, W. Zhang, Y. Hu, T. He, et al., ADAR1 regulates ARHGAP26 gene expression through RNA editing by disrupting miR-30b-3p and miR-573 binding. RNA 19 (2013) 1525-36. doi: 10.1261/rna.041533.113

11. G. Velazquez-Torres, E. Shoshan, C. Ivan, L. Huang, E. Fuentes-Mattei, H. Paret, et al., A-to-I miR-378a-3p editing can prevent melanoma progression via regulation of PARVA expression. Nat Commun 9 (2018) 461. doi: 10.1038/s41467-018-02851-7

12. E.Y. Levanon, M. Hallegger, Y. Kinar, R. Shemesh, K. Djinovic-Carugo, G. Rechavi, et al., Evolutionarily conserved human targets of adenosine to inosine RNA editing. Nucleic Acids Res 33 (2005) 1162-8. doi: 10.1093/nar/gki239

13. M. Nakano, T. Fukami, S. Gotoh, and M. Nakajima, A-to-I RNA Editing Up-regulates Human Dihydrofolate Reductase in Breast Cancer. J Biol Chem 292 (2017) 4873-4884. doi: 10.1074/jbc.M117.775684

14. V. Cesarini, D.A. Silvestris, V. Tassinari, S. Tomaselli, S. Alon, E. Eisenberg, et al., ADAR2/miR-589-3p axis controls glioblastoma cell migration/invasion. Nucleic Acids Res 46 (2018) 2045-2059. doi: 10.1093/nar/gkx1257

15. M. Nakano, T. Fukami, S. Gotoh, M. Takamiya, Y. Aoki, and M. Nakajima, RNA Editing Modulates Human Hepatic Aryl Hydrocarbon Receptor Expression by Creating MicroRNA Recognition Sequence. J Biol Chem 291 (2016) 894-903. doi: 10.1074/jbc.M115.699363

16. T.H. Chan, A. Qamra, K.T. Tan, J. Guo, H. Yang, L. Qi, et al., ADAR-Mediated RNA Editing Predicts Progression and Prognosis of Gastric Cancer. Gastroenterology 151 (2016) 637-650 e10. doi: 10.1053/j.gastro.2016.06.043

17. C. Rossetti, E. Picardi, M. Ye, G. Camilli, A.M. D'Erchia, L. Cucina, et al., RNA editing signature during myeloid leukemia cell differentiation. Leukemia (2017). doi: 10.1038/leu.2017.134

18. L. Chen, Y. Li, C.H. Lin, T.H. Chan, R.K. Chow, Y. Song, et al., Recoding RNA editing of AZIN1 predisposes to hepatocellular carcinoma. Nat Med 19 (2013) 209-16. doi: 10.1038/nm.3043

19. E.M. Amin, Y. Liu, S. Deng, K.S. Tan, N. Chudgar, M.W. Mayo, et al., The RNA-editing enzyme ADAR promotes lung adenocarcinoma migration and invasion by stabilizing FAK. Sci Signal 10 (2017). doi: 10.1126/scisignal.aah3941

20. M.A. Zipeto, A.C. Court, A. Sadarangani, N.P. Delos Santos, L. Balaian, H.J. Chun, et al., ADAR1 Activation Drives Leukemia Stem Cell Self-Renewal by Impairing Let-7 Biogenesis. Cell Stem Cell 19 (2016) 177-191. doi: 10.1016/j.stem.2016.05.004

21. F. Galeano, C. Rossetti, S. Tomaselli, L. Cifaldi, M. Lezzerini, M. Pezzullo, et al., ADAR2-editing activity inhibits glioblastoma growth through the modulation of the CDC14B/Skp2/p21/p27 axis. Oncogene 32 (2013) 998-1009. doi: 10.1038/onc.2012.125

22. E. Shoshan, A.K. Mobley, R.R. Braeuer, T. Kamiya, L. Huang, M.E. Vasquez, et al., Reduced adenosine-to-inosine miR-455-5p editing promotes melanoma growth and metastasis. Nat Cell Biol 17 (2015) 311-21. doi: 10.1038/ncb3110

23. G. Nigita, M. Acunzo, G. Romano, D. Veneziano, A. Laganà, M. Vitiello, et al., microRNA editing in seed region aligns with cellular changes in hypoxic conditions. Nucleic Acids Research 44 (2016) 6298-6308. doi: 10.1093/nar/gkw532

24. Y. Pinto, I. Buchumenski, E.Y. Levanon, and E. Eisenberg, Human cancer tissues exhibit reduced A-to-I editing of miRNAs coupled with elevated editing of their targets. Nucleic Acids Res 46 (2018) 71-82. doi: 10.1093/nar/gkx1176

25. V. Tassinari, V. Cesarini, D.A. Silvestris, and A. Gallo, The adaptive potential of RNA editing-mediated miRNA-retargeting in cancer. Biochim Biophys Acta Gene Regul Mech 1862 (2019) 291-300. doi: 10.1016/j.bbagrm.2018.12.007

26. A. Salameh, A.K. Lee, M. Cardo-Vila, D.N. Nunes, E. Efstathiou, F.I. Staquicini, et al., PRUNE2 is a human prostate cancer suppressor regulated by the intronic long noncoding RNA PCA3. Proc Natl Acad Sci U S A 112 (2015) 8403-8. doi: 10.1073/pnas.1507882112

27. T. Bhalla, J.J. Rosenthal, M. Holmgren, and R. Reenan, Control of human potassium channel inactivation by editing of a small mRNA hairpin. Nat Struct Mol Biol 11 (2004) 950-6. doi: 10.1038/nsmb825

28. A. Beghini, C.B. Ripamonti, P. Peterlongo, G. Roversi, R. Cairoli, E. Morra, et al., RNA hyperediting and alternative splicing of hematopoietic cell phosphatase (PTPN6) gene in acute myeloid leukemia. Hum Mol Genet 9 (2000) 2297-304. doi: 10.1093/oxfordjournals.hmg.a018921

29. T. Shimokawa, M.F. Rahman, U. Tostar, E. Sonkoly, M. Stahle, A. Pivarcsi, et al., RNA editing of the GLI1 transcription factor modulates the output of Hedgehog signaling. RNA Biol 10 (2013) 321-33. doi: 10.4161/rna.23343

30. L. Zhang, C.S. Yang, X. Varelas, and S. Monti, Altered RNA editing in 3' UTR perturbs microRNA-mediated regulation of oncogenes and tumor-suppressors. Sci Rep 6 (2016) 23226. doi: 10.1038/srep23226

31. Y. Choudhury, F.C. Tay, D.H. Lam, E. Sandanaraj, C. Tang, B.T. Ang, et al., Attenuated adenosine-to-inosine editing of microRNA-376a* promotes invasiveness of glioblastoma cells. J Clin Invest 122 (2012) 4059-76. doi: 10.1172/JCI62925

32. C. Anadon, S. Guil, L. Simo-Riudalbas, C. Moutinho, F. Setien, A. Martinez-Cardus, et al., Gene amplification-associated overexpression of the RNA editing enzyme ADAR1 enhances human lung tumorigenesis. Oncogene 35 (2016) 4407-13. doi: 10.1038/onc.2015.469

33. W. Wu, L. Wu, M. Zhu, Z. Wang, M. Wu, P. Li, et al., miRNA Mediated Noise Making of 3'UTR Mutations in Cancer. Genes (Basel) 9 (2018). doi: 10.3390/genes9110545

34. X. Xu, Y. Wang, K. Mojumdar, Z. Zhou, K.J. Jeong, L.S. Mangala, et al., A-to-I-edited miRNA-379-5p inhibits cancer cell proliferation through CD97-induced apoptosis. J Clin Invest 129 (2019) 5343-5356. doi: 10.1172/JCI123396

35. J. Yeo, R.A. Goodman, N.T. Schirle, S.S. David, and P.A. Beal, RNA editing changes the lesion specificity for the DNA repair enzyme NEIL1. Proc Natl Acad Sci U S A 107 (2010) 20715-9. doi: 10.1073/pnas.1009231107

36. L. Goldberg, M. Abutbul-Amitai, G. Paret, and Y. Nevo-Caspi, Alternative Splicing of STAT3 Is Affected by RNA Editing. DNA Cell Biol 36 (2017) 367-376. doi: 10.1089/dna.2016.3575

37. S. Tomaselli, F. Galeano, S. Alon, S. Raho, S. Galardi, V.A. Polito, et al., Modulation of microRNA editing, expression and processing by ADAR2 deaminase in glioblastoma. Genome Biol 16 (2015) 5. doi: 10.1186/s13059-014-0575-z

38. W.H. Liu, C.H. Chen, K.H. Yeh, C.L. Li, Y.J. Wu, D.S. Chen, et al., ADAR2-mediated editing of miR-214 and miR-122 precursor and antisense RNA transcripts in liver cancers. PLoS One 8 (2013) e81922. doi: 10.1371/journal.pone.0081922

39. F. Galeano, A. Leroy, C. Rossetti, I. Gromova, P. Gautier, L.P. Keegan, et al., Human BLCAP transcript: new editing events in normal and cancerous tissues. Int J Cancer 127 (2010) 127-37. doi: 10.1002/ijc.25022
